# Alexithymia and Psychological Distress in Patients With Fibromyalgia and Rheumatic Disease

**DOI:** 10.3389/fpsyg.2019.01735

**Published:** 2019-07-31

**Authors:** Laura Marchi, Francesca Marzetti, Graziella Orrù, Simona Lemmetti, Mario Miccoli, Rebecca Ciacchini, Paul Kenneth Hitchcott, Laura Bazzicchi, Angelo Gemignani, Ciro Conversano

**Affiliations:** Department of Surgical, Medical and Molecular Pathology and Critical Care Medicine, University of Pisa, Pisa, Italy

**Keywords:** chronic pain, fibromyalgia, alexithymia, rheumatoid arthritis, depression, anxiety

## Abstract

**Background:**

Fibromyalgia syndrome (FMS) is a chronic rheumatologic disease characterized by widespread musculoskeletal pain and other psychopathological symptoms which have a negative impact on patients’ quality of life. FMS is frequently associated with alexithymia, a multidimensional construct characterized by difficulty in identifying feelings (DIF) and verbally communicating them difficulty describing feelings (DDF) and an externally oriented cognitive thinking style (EOT). The aim of the present study was to investigate the relationship between alexithymia, anxious and depressive symptoms and pain perception, in patients with FMS and other rheumatic diseases (RD).

**Methods:**

The sample consisted of 127 participants (M = 25, F = 102; mean age: 51.97; SD: 11.14), of which 48 with FMS, 41 with RD and 38 healthy control group (HC). All groups underwent to a test battery investigating anxiety and depressive symptoms (HADS), pain (VAS; QUID-S/-A) and alexithymia (TAS-20).

**Results:**

A high prevalence of alexithymia (TAS ≥ 61) was found in FMS (47.9%) and RD (41.5%) patients, compared to the HC group (2.6%). FMS patients showed significant higher scores than HC on DIF, DDF, EOT, anxiety and depression. The clinical sample, FMS and RD groups combined (*n* = 89), alexithymic patients (AL, *n* = 40) exhibited higher scores in pain and psychological distress compared to non-alexithymic patients (N-AL, *n* = 34). Regression analysis found no relationship between alexithymia and pain in AL, meanwhile pain intensity was predicted by anxiety in N-AL.

**Conclusion:**

While increasing clinical symptoms (pain intensity and experience, alexithymia, anxiety, and depression) in patients with fibromyalgia or rheumatic diseases, correlations were found on the one side, between alexithymia and psychological distress, on the other side, between pain experience and intensity. Meanwhile, when symptoms of psychological distress and alexithymia were subthreshold, correlations with pain experience and intensity became stronger.

## Introduction

Fibromyalgia syndrome (FMS) is a chronic condition characterized by widespread musculoskeletal pain, tenderness of the muscles, stiffness and extra-articular symptoms such as fatigue, anxiety, sleep disorders depression and functional impairment of daily living activities (Gerdle et al., 2008; Jahan et al., 2012). These factors may negatively influence the quality of life of the individuals affected. An increasing number of studies have examined patients’ experiences of illness across a variety of chronic conditions, amongst others, osteoporosis (Catalano et al., 2018; Martino et al., 2018a,b), diabetes (Marchini et al., 2018; Settineri et al., 2019), chronic pain (Catalano et al., 2017) and FMS (Verbunt et al., 2008; Lee et al., 2017) using comprehensive health-related quality of life questionnaires or other outcomes measures provided by the clinical research (Coin et al., 2009) in order to evaluate the burden of diseases, which could be used for treatment planning and monitoring of syndrome progression.

In the context of fibromyalgia, several studies have shown that fibromyalgia is a widespread condition, commonly occurring in young or middle-aged females and the prevalence in the general population is between 0.5 and 5% (Branco et al., 2010).

Despite the increasing knowledge about FMS, the etiopathogenesis of the disease is still unclear. Many factors seem to be involved: genetic, immunological, hormonal, psychological and environmental (Bellato et al., 2012; Albrecht et al., 2019; Atzeni et al., 2019). Central sensitization mechanisms and pain neuromodulation seem to play a primary role in FMS symptoms (Bellato et al., 2012). Among all rheumatic pathologies, FMS is ranked as second for level of diffusion, after osteoarthritis (Clauw, 2014) with a male/female ratio of 1:9 (Bartels et al., 2009). FMS symptoms can vary and are similar to those of several other conditions and the absence of specific laboratory tests or biomarkers make the diagnostic process rather complex. In order to overcome to the mentioned limitations, the American College of Rheumatology (ACR) has approved quantitatively validated classification criteria for FMS which are the most commonly used in clinical and therapeutic research as follows: (1) Generalized pain, defined as pain present in at least 4 of 5 body regions; (2) specific Widespread Pain Index (WPI) and Symptom Severity Scale (SSS): WPI ≥ 7 e SSS ≥ 5 or 6 ≥ WPI ≥ 4 e SSS ≥ 9; (3) symptoms have been present for at least 3 months; (4) FMS diagnosis is valid even in the presence of other diagnoses and doesn’t exclude the presence of other illnesses (Wolfe et al., 2016).

Chronic and diffuse pain seems to be the core symptom of FMS, is associated with a high sensitivity to touch, a lowering of pain threshold, headache, gastrointestinal disorders and severe fatigue (Wallace and Hallegua, 2004; Marcus et al., 2005; Russell and Raphael, 2008; Wolfe and Häuser, 2011; Giamberardino et al., 2016; Doerr et al., 2017). Sleep disorders are also common among FMS patients and are characterized by difficulty in falling asleep, frequent nocturnal awakenings and non-restorative sleep (Palagini et al., 2016; Roth et al., 2016). Furthermore, many studies have reported a high prevalence of cognitive symptoms compared to other rheumatic diseases (RD) (Katz et al., 2004; Gelonch et al., 2017); in particular, a triad of cognitive disturbances (concentration difficulties, short-term memory problems, difficulty in multitasking) and a state of mental confusion termed “fibro-fog” (Katz and Leavitt, 2014) is often present.

Additionally, FMS in females seems to be associated with sexual dysfunctions, such as decreased sexual desire and the presence of pain during sexual intercourse, though psychiatric comorbidity may have more influence on sexual satisfaction than the presence of the rheumatic disease itself (Kalichman, 2009; Yilmaz et al., 2012; Bazzichi et al., 2013; Matarin Jimenez et al., 2017).

Psychiatric disorders such as depression, anxiety, obsessive-compulsive disorder and post-traumatic stress disorder are frequently comorbid with FMS (Henningsen et al., 2003; Dell’Osso et al., 2011; Clauw, 2014; Løge-Hagen et al., 2018; Conversano et al., 2019). The most common comorbidities are mood disorders, with a prevalence of 29–34.8% and anxiety disorders, with a prevalence of 22.3–32.2%; indeed, a careful screening of depressive and anxiety symptoms and their proper management is a primary target in FMS (Epstein et al., 1999; Thieme et al., 2004; Uguz et al., 2010; Piccinni et al., 2011; Consoli et al., 2012; Veltri et al., 2012; Davis et al., 2014; Kudlow et al., 2015). Several studies also highlight the presence of variables associated with psychological vulnerability such as low self-esteem, neuroticism, dependency, passivity, victimization, catastrophization, irritability and maladaptive response to loss (Hassett et al., 2000; Bradley, 2005; Conversano et al., 2010, 2018a,b; Carmassi et al., 2014; Bucourt et al., 2017). Fibromyalgia may indeed negatively affect daily life and mood, consequently inducing feelings of hopelessness, sadness, anger, anxiety or stress. Evidence also suggests that intense negative emotions accompanied by no foreseeable change for the future may lead to mental pain (Verrocchio et al., 2016).

Chronic pain conditions seem to be associated with the presence of alexithymic traits, in particular disorders such as chronic low back pain, FMS, temporomandibular pain and myofascial pain syndrome (Ak et al., 2004; Sayar et al., 2004; Celikel and Saatcioglu, 2006; Lumley et al., 2007; Saariaho et al., 2017). Some studies show that alexithymia is common in FMS patients (Celikel and Saatcioglu, 2006; Taskin et al., 2007; Castelli et al., 2012; Di Tella et al., 2018; Aaron et al., 2019), although others report no differences in alexithymia scores in FMS patients versus healthy controls (Malt et al., 2002). Alexithymia is a complex, multidimensional psychological construct that describes both a difficulty in cognitive processing of emotional experience and a deficit in emotional regulation (Taylor et al., 1997). This construct, which literally means “lack of words to describe emotions,” was first introduced by Sifneos in 1972 to describe people who were unable to communicate their feelings and had poor imaginative abilities and who often presented a series of somatic symptoms. The main aspects of alexithymia are the following: difficulty identifying and describing emotions and feelings, difficulty distinguishing between emotions and body associated sensations and a cognitive style which is practically oriented, with a paucity of imaginative processes (Taylor et al., 1997). High levels of alexithymia seem to prevent a correct emotional regulation, especially regarding negative emotions, and to cause a chronic physiological hyperactivation, physical symptoms and somatic amplifications (Lumley et al., 1996). Individuals showing high levels of alexithymia not only have limited ability to reflect and regulate their emotions, but also struggle to verbally communicate them (Taylor, 2000; Torrado et al., 2018).

The theoretical stance that alexithymia is a personological trait characterized by a deficit of regulation and emotional processing, has made this construct particularly useful in exploring the role of personality and emotions in the pathogenesis of different somatic diseases (Taylor, 2000). Associations between alexithymia and different somatic pathologies such as chronic intestinal inflammatory diseases (Porcelli et al., 1996; Mazaheri et al., 2012), chronic respiratory disorders (Serrano et al., 2006; Baiardini et al., 2011), dermatological conditions (Willemsen et al., 2008; Talamonti et al., 2016) and neuromuscular pathologies (Hosoi et al., 2010) are reported frequently in the literature. However, it has been highlighted that alexithymia can also manifest as a transient state that varies in severity in accordance with stress levels and the presence of psychopathological conditions, including depression and anxiety disorders (Honkalampi et al., 2000; Pollatos et al., 2011; Montoro et al., 2016).

Overall the presence of alexithymia in patients with chronic pain and its influence on pain intensity is still unclear: many studies haven’t found significant associations (Cox et al., 1994; Evren et al., 2006), while others found only weak correlations (Lumley et al., 2005a,b; Celikel and Saatcioglu, 2006) and in some studies this relationship appeared to be mediated by negative affect, especially depression (Di Tella and Castelli, 2016; Aaron et al., 2019). Castelli et al. (2012) have shown positive associations between alexithymia and the affective experience of pain, anxiety, depression, QoL and neuroticism. A recent study (Di Tella et al., 2017) has shown the presence of higher levels of pain symptoms and psychological distress in alexithymic FMS patients compared to non-alexithymic ones. Moreover, the results of the study highlighted a positive association between alexithymia, particularly between difficult in identifying feelings, and the affective dimension of pain experience, supporting the hypothesis that in patients with chronic muscular disease, alexithymia is more related to the unpleasant affective dimension of pain than the sensorial one. The association between alexithymia and pain was specifically mediated by anxiety.

Several studies have pointed out that differences in levels of alexithymia of FMS and healthy controls significantly decrease or disappear once anxiety and depression are statistically controlled, supporting the view that alexithymia may represent a state phenomenon that varies in relation to severity of clinical symptoms (Steinweg et al., 2011; Marchesi et al., 2014; Montoro et al., 2016). Montoro et al. (2016) showed that alexithymia was more closely associated with clinical variables (pain, fatigue, sleep, anxiety, depression, QoL) in healthy subjects than in FMS group in which many associations disappeared when anxiety and depression were controlled.

Therefore, the precise role of alexithymia in fibromyalgia has not yet been clarified. The present work aims to investigate and clarify the relationship between alexithymia, pain, anxiety and depression in FMS patients. The first aim is to further evaluate alexithymia in FMS patients, in comparison with healthy subjects and patients with other chronic pain pathologies, such as arthritis, and to explore its relationship with pain perception and other psychological factors, such as emotional distress (anxiety and depression). The second aim is to verify whether alexithymia is related to pain perception (in its sensory and affective components) and pain intensity, and to what extent psychological distress, especially anxiety and depression, is involved in this relationship.

According to previous researches, we hypothesized that FMS and RD patients would exhibit higher levels of alexithymia, pain perception, anxiety and depression while compared with HC. We also hypothesized that alexithymia might influence pain affective dimension, as it might be a state phenomenon that varies in relation to severity of anxious and depressive symptoms.

## Materials and Methods

### Participants’ Inclusion/Exclusion Criteria

One hundred and twenty-seven subjects with a mean age of 51.97 years (SD = 11.14) were recruited for the study. Chronic pain patients were recruited between May and December 2018 at the University of Pisa Hospital’s Rheumatology Unit (A.O.U.P). The clinical sample consisted of two groups: patients with a diagnosis of either FMS (*n* = 48), according the ACR criteria (Wolfe et al., 2011), or rheumatic disease (RD; *n* = 41). All patients had a main diagnosis, made by an expert rheumatologist. The exclusion criteria for both groups were: age below 18, education level below 5 years, insufficient knowledge of Italian language and presence of an important neurological and/or psychiatric disorder.

The healthy control group (HC; *n* = 38) was recruited from patients’ partners or relatives that accompanied them to visit. They had no history of chronic pain and met the same exclusion criteria.

### Ethics

The study was approved by Hospital of Pisa Ethics Committee and was conducted in accordance with the Declaration of Helsinki. All participants gave their written informed consent before participating in the study.

### Materials

#### Alexithymia

The Italian version of the Toronto Alexithymia Scale (TAS-20; Bressi et al., 1996) was used to assess alexithymia. This is a self-administered questionnaire consisting of 20 items scored on a 5-point Likert scale recording respondents degree of agreement/disagreement for each statement (1 = I don’t agree at all; 2 = I don’t agree very much; 3 = I’m not either neither agree nor disagree; 4 = I agree in part; 5 = I completely agree). TAS-20 has three subscales representing three main facets of alexithymia: the Difficulty Identifying Feelings (DIF, seven items) subscale, measures difficulties in distinguishing between specific emotions and/or bodily sensations related to emotional arousal; the Difficulty Describing Feelings (DDF, five items) subscale, indicates inability to verbalize one’s experienced emotions; the Externally Oriented Thinking (EOT, eight items) subscale, indicates the tendency to focus attention externally instead of considering inner emotional experience. A global score ranging between 20 and 100 is calculated as a sum of the three subscales and there are cut-off points as following: alexithymia, TAS ≥ 61; borderline, 52 < TAS < 61; no alexithymia, TAS ≤ 51. It is the most widely used instrument both in clinical and research for the evaluation of alexithymia and it has good internal consistency (Cronbach’s alpha = 0.81) and good test–retest reliability (0.77, *p* < 0.01) (Bagby et al., 1994a,b; Parker et al., 2003; Taylor et al., 2003).

#### Pain Evaluation

##### Pain intensity

The Visual Analog Scale for Pain was used to assess pain intensity (VAS; McCormack et al., 1988). The VAS is a single item scale and consists of a 10 cm horizontal line on which the subject had to indicate the average intensity of pain experienced in the previous week: the scale ranges from 0 (“no pain”) to 10 (“extreme pain”). VAS is widely used due to its simplicity and adaptability to various settings and populations; it has been used in different populations, including those with RD (Huskisson, 1974; Downie et al., 1978). The scale showed good statistical qualities both for the evaluation of chronic pain and for experimental scopes (Price et al., 1983), Test–retest reliability has been shown to be good (*r* = 0.94, *p* < 0.001) (Ferraz et al., 1990).

##### Pain experience

The Questionario Italiano sul Dolore, the Italian adaptation of the McGill Pain Questionnaire (Melzack, 1975) was administered to evaluate pain experience (QUID; De Benedittis et al., 1988). It is a self-administered questionnaire that asks subjects to choose adjectives from 16 subclasses to describe the pain experienced during the previous month. These adjectives belong to four categories: sensory, which describes pain in terms of spatial and temporal properties; affective, referred to the affective qualities of pain such as tension, fear, autonomic reactions; evaluative, which reports the overall subjective impression on the painful experience and mixed that combines different sensory, affective and evaluative aspects. Only the sensory (QUID-S) and affective (QUID-A) components of pain experience were considered for the purposes of the present study. Test–retest reliability in population with a variety of conditions including arthritis and other musculoskeletal conditions was good (*r* = 0.70) (Melzack, 1975; Love et al., 1989; Broderick et al., 2008).

#### Psychological Distress

To assess symptoms of psychological distress, the Italian adaptation of the Hospital Anxiety and Depression Scale was used (HADS; Zigmond and Snaith, 1983; Costantini et al., 1999). This instrument consists of 14 items scored on a 3-point Likert scale divided into two subscales: anxiety (HADS-A) and depression (HADS-D), both composed of seven items. Subscales score ranges from 0 to 21 and cut-off points are the following: normal, HADS-A or HADS-D ≤ 7; borderline/abnormal level of anxiety or depression, 8 ≤ HADS-A or HADS-D ≤ 10; clinically relevant level of depression or anxiety, 11 ≤ HADS-A or HADS-D ≤ 21 (Zigmond and Snaith, 1983). Cronbach’s alpha for HADS-A varied from 0.68 to 0.93 (mean 0.83) and for HADS-D from 0.67 to 0.90 (mean 0.82) (Bjelland et al., 2002).

### Statistical Analysis

Distributions were assessed using the Kolmogorov–Smirnov test. All clinical variables for FMS, RD and HC groups were not normally distributed, and so the data were analyzed using Kruskal-Wallis with Dunn’s multiple comparisons. Spearman’s correlation was computed to evaluate the relationship between alexithymia, pain perception and intensity, anxiety and depression. Partial correlations were performed to control anxiety and depression effects. All clinical variables were normally distributed in the Alexithymia and No Alexithymia subgroups: *t*-test for independent samples was used to assess differences between subgroups. Pearson’s coefficient was computed to evaluate variables correlations and multivariate linear regression was performed. *P* values <0.05 were considered statistically significant. The analysis was conducted using IBM SPSS statistics version 25.

## Results

### Demographic Characteristics and Group Differences in Clinical Variables

A sample of 48 patients with FMS diagnosis (M = 1; age mean: 52.94; SD: 11.50), 41 patients with rheumatic disease diagnosis (M = 10; age mean: 54.74; SD:10.77) and a control group of 38 healthy subjects (M = 14; age mean: 47.92; SD:10.15) were included in the present study. Demographic characteristics are summarized in Table 1.

**TABLE 1:** Demographic characteristics and prevalence in FMS, RD and HC groups.

	**FMS group**			**RD group**			**HC group**		
	***n* = 48**			***n* = 41**			***n* = 38**		
	**Mean (SD)**	**Range**	***n* (%)**	**Mean (SD)**	**Range**	***n* (%)**	**Mean (SD)**	**Range**	***n* (%)**
Age	52.94 (11.50)	20–72		54.74 (10.77)	25–73		47.92 (10.15)	22–73	
**Gender**									
F			47 (97.9%)			31 (75.6%)			24 (63.2%)
M			1 (2.1%)			10 (24.4%)			14 (36.8%)
Education (years)	10.85 (3.15)	5–18		10.75 (4.03)	5–18		13.23 (4.19)	5–18	
Duration of illness (years)	9.02 (6.15)	1–28		11.64 (8.59)	1–36		–		
**Alexithymia**									
Alexithymic			23 (47.9%)			17 (41.5%)			1 (2.6%)
Non-Alexithymic			17 (35.4%)			17 (41.5%)			33 (86.8%)
Borderline			8 (16.7%)			7 (17.1%)			4 (10.5%)
**Psychological distress**									
HADS-A ≥ 8			28 (58.3%)			20 (48.8%)			2 (5.3%)
HADS-D ≥ 8			32 (66.7%)			21 (51.2%)			5 (13.2%)

*FMS; fibromyalgia, RD; rheumatic disease diagnosis, HC; healthy subjects, HADS-A; Hamilton Anxiety and Depression Scale-Anxiety, HADS-D; Hamilton Anxiety and Depression Scale-Depression.*

TAS ≥ 61 was found in FMS (47.9%) and RD (41.5%) patients, compared to the HC group (2.6%). Subjects in the FMS group reported significant higher scores than HC on TAS total score (*p* < 0.0001) (Table 2): particularly, FMS patients have higher scores on DIF (*p* < 0.0001), DDF (*p* < 0.0003) and EOT (*p* < 0.01) than healthy subjects. The same results were found comparing RD vs. HC groups.

**TABLE 2:** Questionnaire scales and subscales scores.

	**FMS group**	**RD group**	**HC group**	
	***n* = 48**	***n* = 41**	***n* = 38**	
	**Median (interquartile range)**			***P*-value**
**Alexithymia-TAS-20**				
DIF	22.00 (14.75–26.00)	17.00 (14.00–23.00)	11.00 (7.50–14.00)	< 0.0001
DDF	16.50 (10.75–19.00)	15.00 (12.00–17.00)	11.00 (8.00–14.75)	0.001
EOT	19.00 (15.75–22.00)	20.00 (16.00–22.00)	16.00 (14.00–18.00)	0.003
TAS	60.00 (46.75–68.00)	56.00 (47.00–64.00)	40.50 (32.25–48.00)	< 0.0001
**Pain-QIUD**				
QUID-S	0.33 (0.23–0.42)	0.30 (0.12–0.39)	0.03 (0.00–0.14)	< 0.0001
QUID-A	0.40 (0.27–0.53)	0.20 (0.07–0.40)	0.00 (0.00–0.18)	< 0.0001
VAS	7.00 (4.75–8.00)	5.00 (4.00–7.00)	1.00 (0.00–4.00)	< 0.0001
**Psychological distress**				
HADS-A	9.00 (6.00–13.00)	7.00 (4.00–11.00)	4.00 (2.00–5.00)	< 0.0001
HADS-D	9.00 (6.75–11)	8.00 (5.00–10.00)	3.00 (1.00–5.75)	< 0.0001

*TAS-20; Toronto Alexithymia Scale-20 Item Version, DIF; Difficulty Identifying Feelings, DDF; Difficulty Describing Feelings, EOT; Externally-Oriented Thinking, TAS; TAS Total Score, QUID; Questionario Italiano sul Dolore, Sensorial Pain Rating Index (QUID-S), Affective Pain Rating Index (QUID-A), VAS; Visual Analog Scale for pain intensity, QUID-T; Total Pain Index, HADS-A; Hamilton Anxiety and Depression Scale-Anxiety, HADS-D; Hamilton Anxiety and Depression Scale-Depression. *P* values from Kruskal-Wallis non-parametric test.*

58.3% of FMS patients and 48.8% of RD patients showed higher scores on HADS-A (HADS-A ≥ 8) and 66.7% of FMS patients and 51.2% of RD patients reported high scores on HADS-D (HADS-D ≥ 8) while only 5.3 and 13.2% of HC, reported scores above the cut-off on HADS-A and HADS-D, respectively. Patients in the FMS group showed significant higher scores than HC on HADS-A (*p* < 0.0001) and HADS-D (*p* < 0.0001), in the same way as RD patients did compared to HC (HADS-A, *p* < 0.0001; HADS-D, *p* < 0.0001). Differences in alexithymia and psychological distress did not differ between the FMS and RD groups.

Questionario Italiano sul Dolore subscales (QUID-S, QUID-A) and VAS scores were significantly higher in subjects with FMS than HC. Moreover, FMS patients experienced a higher pain intensity (*p* = 0.05) than RD patients that is more characterized by the affective dimension of pain (*p* = 0.013).

### Association Between Alexithymia, Pain and Psychological Distress in FMS, RD, and HC Groups

Correlation analysis conducted between alexithymia and other variables in FMS group (Table 3) showed a significant moderate positive association between DIF subscale and HADS-A (*r*_s_ = 0.387, *p* = 0.007). In this group, years of education was negatively correlated with DIF (*r*_s_ = −0.331, *p* = 0.022) and DDF (*r*_s_ = −0.316, *p* = 0.029) and both relationships were maintained while controlling for anxiety and depression. No significant correlations between alexithymia and pain were observed, meanwhile pain and psychological distress showed significant correlations.

**TABLE 3:** Significant Spearman correlation coefficients: associations between alexithymia and questionnaire scores in patients with fibromyalgia (FMS), rheumatic diseases (RD) and healthy subjects (HC).

	**FMS group**	**RD group**	**HC group**
	***n* = 48**	***n* = 41**	***n* = 38**
**DIF**			
QUID-S	0.261	**0.453^∗∗^**	**0.445^∗∗^**
QUID-A	0.111	**0.466^∗∗^**	**0.368^∗^**
VAS	0.163	**0.480^∗∗^**	**0.505^∗∗∗^**
HADS-A	**0.387^∗∗^**	**0.383^∗^**	**0.493^∗∗^**
HADS-D	0.264	0.151	**0.411^∗^**
**DDF**			
QUID-S	0.211	0.296	0.239
QUID-A	–0.022	0.143	0.295
VAS	0.144	0.295	**0.322^∗^**
HADS-A	0.076	0.055	0.243
HADS-D	0.255	0.094	**0.504^∗∗∗^**
**EOT**			
QUID-S	0.275	–0.147	**0.358^∗^**
QUID-A	0.209	–0.167	**0.380^∗^**
VAS	0.134	0.195	0.281
HADS-A	0.238	0.225	–0.006
HADS-D	0.102	**0.341^∗^**	0.248

*Same abbreviations as Table 2. Bold data indicate correlations that are significant. ^∗^*p* < 0.05; ^∗∗^*p* < 0.01; ^∗∗∗^*p* < 0.001.*

Patients with RD exhibited significant moderate positive correlations between DIF and the QUID-S (*r*_s_ = 0.453, *p* = 0.003), QUID-A (*r*_s_ = 0.466, *p* = 0.002), VAS (*r*_s_ = 0.480, *p* = 0.002) and HADS-A (*r*_s_ = 0.383, *p* = 0.014). Furthermore, EOT was positively associated with HADS-D scores (*r*_s_ = 0.341, *p* = 0.030). Psychological distress and pain showed significant correlations. While controlling for anxiety and depression in RD group, all the positive correlations between DIF and QUID-S and QUID-A were maintained. No significant associations with demographical variables such as age, years of education or duration of illness were observed in this group.

Alexithymia was more closely associated with psychological distress and pain in healthy subjects: DIF positively correlated with QUID-S (*r*_s_ = 0.445, *p* = 0.024), QUID-A (*r*_s_ = 0.368, *p* = 0.017), VAS (*r*_s_ = 0.505, *p* = 0.001), HADS-A (*r*_s_ = 0.493, *p* = 0.002) and HADS-D (*r*_s_ = 0.411, *p* = 0.011); DIF scores correlated with VAS (*r*_s_ = 0.322, *p* = 0.050) and HADS-D (*r*_s_ = 0.504, *p* = 0.001) while EOT is associated with QUID-S (*r*_s_ = 0.358, *p* = 0.028), QUID-A (*r*_s_ = 0.380, *p* = 0.019) and age (*r*_s_ = 0.420, *p* = 0.009). When anxiety and depression were controlled, positive correlations between DIF and VAS and between EOT and age were maintained.

### Differences in Demographic Characteristics and Clinical Variables Between Patients With Alexithymia vs. No Alexithymia

In a clinical sample, the FMS and RD groups combined (*n* = 89), the total TAS score showed that 44.9% (40/89) patients had alexithymia (AL; TAS score ≥61), 38.2% (34/89) patients did not have aleithymia (N-AL; TAS-20 score ≤51) and 16.8% (15/89) patients were borderline (52 < TAS-20 < 61). Patients in the borderline group were excluded. AL and N-AL groups had no significant differences in age, education and duration of illness (Table 4).

**TABLE 4:** Demographic characteristics and prevalence in alexithymia vs. no alexithymia groups.

	**Alexithymia**			**No alexithymia**		
	***n* = 40**			***n* = 34**		
	**Mean (SD)**	**Range**	***n* (%)**	**Mean (SD)**	**Range**	***n* (%)**
Age	52,85 (12,67)	20–72		55.16 (8.99)	27–73	
**Gender**						
F			34 (85.0%)			32 (94.1%)
M			6 (15.0%)			2 (5.9%)
Education (years)	10.15 (3.64)	5–18		11.73 (3.63)	5–18	
Duration of illness (years)	9.06 (6.30)	1–24		12.00 (9.18)	1–36	
**Psychological distress**						
HADS-A ≥ 8			27 (67.5%)			14 (41.2%)
HADS-D ≥ 8			28 (70.0%)			16 (47.1%)

*HADS-A; Hamilton Anxiety and Depression Scale-Anxiety, HADS-D; Hamilton Anxiety and Depression Scale-Depression.*

Patients with alexithymia showed significant higher scores on HADS-A (*p* = 0.004), HADS-D (*p* = 0.007), VAS (*p* = 0.005) and QUID-S (*p* = 0.003) (Table 5).

**TABLE 5:** Questionnaire scales and subscales scores in alexithymia vs. no alexithymia groups.

	**Alexithymia**	**No alexithymia**	***F***	***P* values**
	***n* = 40**	***n* = 34**		
		
	**Mean (SD)**			
**Pain**				
QUID-S	0.34 (0.14)	0.24 (0.14)	0.043	**0.003**
QUID-A	0.37 (0.20)	0.28 (0.23)	0.881	0.064
VAS	6.75 (1.99)	5.18 (2.62)	3.147	**0.005**
**Psychological distress**				
HADS-A	10.00 (4.42)	7.15 (3.69)	0.841	**0.004**
HADS-D	9.28 (3.30)	7.03 (3.61)	0.466	**0.007**

*Same abbreviations as Table 2. *P* values from *t*-test for independent samples.*

### Association Between Alexithymia, Pain and Psychological Distress in Patients With Alexithymia vs. No Alexithymia

Correlation analysis (Table 6) in the AL group showed that DIF and DDF were associated with HADS-A (*p* = 0.020) and HADS-D (*p* = 0.027), respectively. Meanwhile, VAS was related to the QUID-S (*p* < 0.0001) and QUID-A (*p* = 0.009) and these relationships were maintained when anxiety and depression effects were controlled. No significant relationship was found between alexithymia and pain in AL group, nevertheless, it was found in N-AL group (Table 7): EOT was positive associated with VAS (*p* = 0.049) and there’s a trend of correlation between DIF and QUID-S (*p* = 0.066). DIF was negatively correlated with QUID-A (*p* = 0.05). Concerning psychological distress, HADS-A was negatively related to DIF (*p* = 0.010) and positively with EOT (*p* = 0.015); HADS-D was positively correlated with EOT (*p* = 0.033). All relationships were moderate.

**TABLE 6:** Significant Pearson correlation coefficients *r*: associations between alexithymia, psychological distress and pain in patients with alexithymia (*n* = 40).

		**1**	**2**	**3**	**4**	**5**	**6**	**7**	**8**
1	DIF	**–**							
2	DDF	**0.381^∗^**	**–**						
3	EOT	**−0.318^∗^**	–0.046	**–**					
4	HADS-A	**0.366^∗^**	–0.108	–0.004	**–**				
5	HADS-D	0.064	**0.350^∗^**	0.089	**0.319^∗^**	**–**			
6	QUID-S	0.173	0.041	–0.230	0.152	0.237	**–**		
7	QUID-A	0.147	–0.084	–0.148	0.196	–0.051	**0.755^∗∗^**	**–**	
8	VAS	0.169	0.199	–0.136	0.267	0.247	**0.685^∗∗^**	**0.407^∗∗^**	**–**

*Same abbreviations as Table 2. Bold data indicate correlations that are significant. ^∗^*p* < 0.05; ^∗∗^*p* < 0.001.*

**TABLE 7:** Significant Pearson correlation coefficients r: associations between alexithymia, psychological distress and pain in non-alexithymic patients (*n* = 34).

		**1**	**2**	**3**	**4**	**5**	**6**	**7**	**8**
1	DIF	**–**							
2	DDF	0.079	**–**						
3	EOT	–0.155	–0.170	**–**					
4	HADS-A	0.160	**−0.438^∗∗^**	**0.415^∗^**	**–**				
5	HADS-D	0.039	–0.318	**0.367^∗^**	**0.685^∗∗∗^**	**–**			
6	QUID-S	**0.404^∗^**	–0.098	–0.031	0.172	0.003	**–**		
7	QUID-A	0.273	**−0.333^∗^**	0.093	0.295	0.212	**0.835^∗∗∗^**	**–**	
8	VAS	0.319	–0.283	**0.340^∗^**	**0.668^∗∗∗^**	**0.451^∗∗∗^**	0.323	**0.377^∗^**	**–**

*Same abbreviations as Table 2. Bold data indicate correlations that are significant. ^∗^*p* < 0.05; ^∗∗^*p* < 0.01; ^∗∗∗^*p* < 0.001.*

In N-AL group, a significant regression model was obtained for VAS as the dependent variable and DIF, EOT, HADS-A, HADS-D and QUID-A as predictors, with a good level of fit with the data (*R*^2^ = 0.53) (Table 8). HADS-A positively predict VAS (β = 0.381, *p* = 0.009). In AL group, VAS was predicted by QUID-S (β = 12.636, *p* < 0.0001) and the regression model explained the 50% of the variance of the data.

**TABLE 8:** Results of multiple regression analysis predicting pain intensity (VAS) from DIF, EOT, HADS-A, HADS-D in alexithymic (*n* = 40) and non-alexithymic patients (*n* = 34).

**Dependent variables**	**Predictors**	**β**	**S.E.**	**Lim. inf. (95%)**	**Lim. sup. (95%)**	***P* values**
**Alexithymia**						
VAS	Intercept	3.415	0.612	2.174	4.656	< **0.0001^∗∗^**
	QUID-S	12.636	2.557	7.455	17.816	< **0.0001^∗∗^**
	QUID-A	–2.532	1.756	–6.089	1.025	0.158
**No alexithymia**						
VAS	Intercept	–1.760	2.206	–6.280	2.759	0.432
	DIF	0.167	0.109	–0.057	0.390	0.137
	EOT	0.089	0.095	–0.105	0.283	0.357
	HADS_A	0.381	0.136	0.103	0.660	**0.009^∗^**
	HADS_D	–0.006	0.131	–0.274	0.262	0.965
	QUID-A	1.686	1.592	–1.575	4.948	0.299

*DIF, Difficulty Identifying Feelings; EOT, Externally-Oriented Thinking; HADS-A, Hamilton Anxiety and Depression Scale-Anxiety; HADS-D, Hamilton Anxiety and Depression Scale-Depression; β, unstandardized beta; S.E., standard error. Bold data indicate significant variables in regression model. ^∗^*p* < 0.01; ^∗∗^*p* < 0.001.*

## Discussion

The main purpose of the research was to investigate the prevalence of psychological distress and alexithymia in fibromyalgia patients, compared to those suffering from arthritis and healthy controls. Moreover, we aimed to describe the role of these variables in pain perception (affective and sensory dimensions) and pain intensity.

The results showed higher levels of alexithymia in patients with fibromyalgia, arthritis and other painful rheumatic conditions compared with healthy controls, according to previous research (Steinweg et al., 2011; Baeza-Velasco et al., 2012; Montoro et al., 2016; Di Tella et al., 2017). Alexithymia scores were higher in all three TAS-20 dimensions. These results may highlight the presence of a greater difficulty in identifying and expressing emotions and an external oriented cognitive style in patients with chronic pain conditions compared to general population. No significant differences were found between the FMS and RD groups, suggesting that both chronic pain conditions may have similar impairment in awareness and emotional regulation.

In regard to psychological distress, we found comorbidities between painful chronic conditions and anxious or depressive clinical symptoms, matching previous studies (Dell’Osso et al., 2011; Clauw, 2014; Løge-Hagen et al., 2018; Conversano et al., 2019). Our clinical groups exhibited a greater psychological impairment compared to healthy controls, with higher levels of anxiety and depression, similarly to the ones reported in Castelli et al. (2012); 60% prevalence of depression (HADS-D) and a 52.7% of anxiety (HADS-A) in FMS group). Despite other studies reporting higher levels of depressive symptoms and psychological distress in the FMS group compared to other chronic rheumatologic disorders (Ozcetin et al., 2007; Piccinni et al., 2011; Scheidt et al., 2014), our findings showed that patients with FMS and RD displayed similar anxiety and depression levels. These discrepancies in results may be due to differences in samples characteristics, for example fibromyalgia patients treated with antidepressant therapies that have relieved clinical symptoms or to the use of different scales to measure psychological variables.

In agreement with Scheidt et al. (2014) results, fibromyalgia patients showed higher levels of pain intensity than RD patients. Moreover, FMS patients had higher scores than RD patients in the affective component of pain, in agreement with Di Tella et al. (2017). Both results are in consistent with the phenomenon of allodynia and pain sensitivity of the FMS syndrome: while FMS patients experienced an increased pain response to non-painful stimuli due to central sensitization, their pain perception seems to be more related to fear, tension and autonomic reaction to stimuli than RD patients.

Concerning associations between alexithymia and pain or psychological distress in FMS group, we found that alexithymia was not related to pain experience or intensity. This result suggest that lower emotional awareness does not play a primary role in pain perception, and it might be a transitional state related to severity of psychological distress, as other authors already highlighted (Steinweg et al., 2011; Marchesi et al., 2014; Montoro et al., 2016). In line with that, a significant relationship was found between DIF and anxiety, confirming the strong association between alexithymia and psychological distress in FMS. Moreover, pain experience and psychological distress had a significant relationship in the FMS group and this result is in line with previous research (Van Houdenhove and Luyten, 2006; Homann et al., 2012). In the light of these findings, we could hypothesize that in patients with FMS anxiety and depression affect both pain experience and emotional awareness and therefore they should be identified and taken into account in the treatment.

Strong correlations between alexithymia, pain and psychological distress were found in RD group. The positive correlation between difficulty in identifying feelings and the different components of pain was maintained, even after anxiety and depression was controlled. This finding suggests that in RD patients, alexithymia plays a more important role in influencing pain, regardless of the presence and severity of comorbid anxiety or depression.

With the aim of exploring the potential impact of alexithymia on the intensity and perception of pain, the clinical group (FMS + RD) was divided into patients with or without alexithymia, according to TAS-20 cut-off scores. Alexithymic patients (AL) had higher scores on anxiety, depression, pain intensity and perception than non-alexithymic ones (N-AL), according to previous studies (Makino et al., 2013; Saariaho et al., 2013; Di Tella et al., 2017). Two different regression models were obtained for AL vs. N-AL groups. In AL group, pain intensity was positively predicted by the sensory dimension of pain. In contrast with previous researches (Di Tella et al., 2017) our sample of patients experienced higher pain intensity that appeared not to be related with psychological distress and alexithymia, but only with the sensory dimension of pain (periodic, pulsing, pounding, penetrating, burning, smarting, tender). Meanwhile, in this group, the relationship between psychological distress and alexithymia was confirmed, suggesting that alexithymia in chronic pain patients might be a state that arise from other psychological conditions (i.e., high levels of depression or anxiety). In the N-AL group instead, anxiety positively predicted pain intensity, confirming the association between psychological distress, alexithymia and pain whereas variables scores are subthreshold. These results were also confirmed in healthy subjects, where strong correlations between al clinical variables were found.

Our results suggest that while clinical symptoms (pain intensity and experience, alexithymia, anxiety and depression) are increased in patients with fibromyalgia or RD, the relationship between pain and other variables disappeared. Two separated blocks of significant correlations appeared: on the one side, pain perception and pain experience are associated, on the other side alexithymia is related to negative affects states. This result also suggest that alexithymia may be a state phenomenon that arise from psychological distress and might be investigated with different scales or within longitudinal studies. Meanwhile, when symptoms of psychological distress and alexithymia were subthreshold, correlations with pain experience and intensity became stronger.

### Limitations

The present study has some limitations that should be considered. The cross-sectional nature of the study does not allow inferences about the causal relations among the variables. Prospective studies could clarify the relation between alexithymia, pain and emotional distress and its trend over time. Moreover, the use of a self-report measure (TAS-20) to assess alexithymia may not have investigated this construct properly, since it requires subjects to identify and describing affects, which is compromised in alexithymic subjects. The future research could use clinical interviews to assess alexithymia. Moreover, the use of a small sample did not permit the generalizability of the results to the fibromyalgia and arthritis patients population.

## Data Availability

The raw data supporting the conclusions of this manuscript will be made available by the authors, without undue reservation, to any qualified researcher.

## Ethics Statement

The studies involving human participants were reviewed and approved by Department of Surgical, Medical and Molecular Pathology, Critical and Care Medicine, University of Pisa. The patients/participants provided their written informed consent to participate in this study.

## Author Contributions

CC conceived the assessment. LM, FM, LB, and CC planned the assessment. LM, FM, and SL contributed to the healthy subjects’ data acquisition. LM, FM, and SL contributed to the patients’ data acquisition. MM and GO contributed to the data analysis. MM, GO, CC, and AG contributed to the data interpretation. LM, FM, GO, RC, PKH, and CC drafted the manuscript. All authors critically revised the manuscript and approved the final version to be published.

## Conflict of Interest Statement

The authors declare that the research was conducted in the absence of any commercial or financial relationships that could be construed as a potential conflict of interest.
